# Supplementary material for: Evaluation of Changes to the Oral Microbiome Based on 16S rRNA Sequencing among Children Treated for Cancer
Source: Cancers (Basel). 2021 Dec 21;14(1):7. doi: 10.3390/cancers14010007 (PMC8750156; doi:10.3390/cancers14010007)
Supplement: Supplementary file 1 [file cancers-14-00007-s001.zip › cancers-1494185-supplementary.pdf]

cancers

MDPI

Supplementary Material: Evaluation of Changes to the Oral Microbiome Based on 16S rRNA Sequencing among Children Treated for Cancer

Patrycja Proc, Joanna Szczepańska, Beata Zarzycka, Małgorzata Szybka, Maciej Borowiec, Tomasz Płoszaj, Wojciech Fendler, Jędrzej Chrzanowski, Małgorzata Zubowska, Małgorzata Stolarska and Wojciech Młynarski

Table S1. Detailed cancer status in the study group.

ID	sex	diagnosis	treatment regimen	age at diagnosis (years)	age at the end of the therapy (years)	length of therapy (months)
18. S.N.	0	Hodgkin's lymphoma	OEPA	13.91	14.33	4
20. W.K.	0	Hodgkin's lymphoma	OEPA	18.25	18.91	8
1. B.B.	1	Hodgkin's lymphoma	OEPA	13.50	16.16	32
11. K.N.	0	Hodgkin's lymphoma	OEPA	15.25	17.66	29
5. D.M.	0	medulloblastoma	I prot CZD	5.83	7.41	19
14. L.K.	1	Ependimoma	VI prot CZD	8.00	9.25	15
3. D.B.	1	Germinoma	V prot CZD	16.00	16.41	5
10. K.M.	1	Germinoma	V prot CZD	12.50	12.91	4
19. T.K.	1	Rhabdomyosarcoma	CWS	16.16	16.75	7
9. K.A.	1	Rhabdomyosarcoma	CWS	6.75	7.83	13
8. K.D	1	non-Hodgkin's lymphoma	B-NHL 13	17.91	18.16	3
16. S.M.	1	non-Hodgkin's lymphoma	B-NHL 13	7.25	9.33	24
4. D.D.	1	acute B-cell lymphoblastic leukemia	B-NHL 13	13.66	14.08	4
6.F.M	1	non-Hodgkin's lymphoma	T-NHL	14.00	16.08	24
2. C.G.	0	neuroblastoma	COJEC/PACE	6.58	9.33	32
13. K.N.	0	acute myeloid leukemia	ANLL 02	13.58	15.08	17
15. R.I.	1	acute lymphoblastic leukemia	ALLIC	16.41	16.83	5
17. S.N.	1	chronic myelogenous leukemia	CML	14.50	17.91	40
12. K.J.	1	tumour of testis	TGM 95	17.66	17.91	3
7.J.W	0	ovarian tumour	TGM 95	10.25	10.91	8

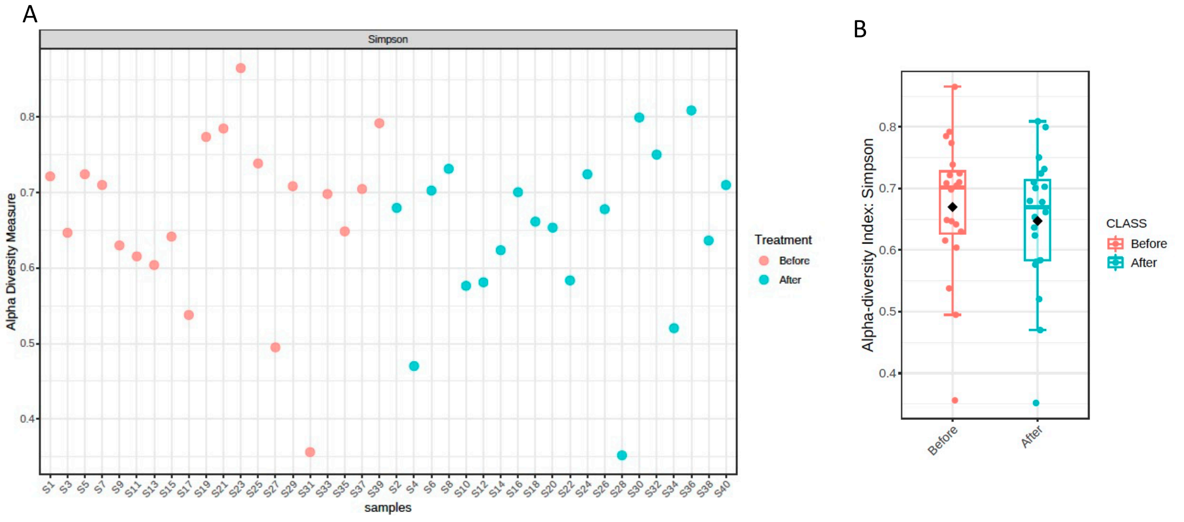

Figure S1. Alpha-diversity measure using Simpson at Genus level across all the samples. A - The samples are represented on X-axis and their estimated diversity on Y-axis, each sample is colored based on Treatment class; B – Box plot presentation, each boxplot represents the diversity distribution of a group present within Treatment class.

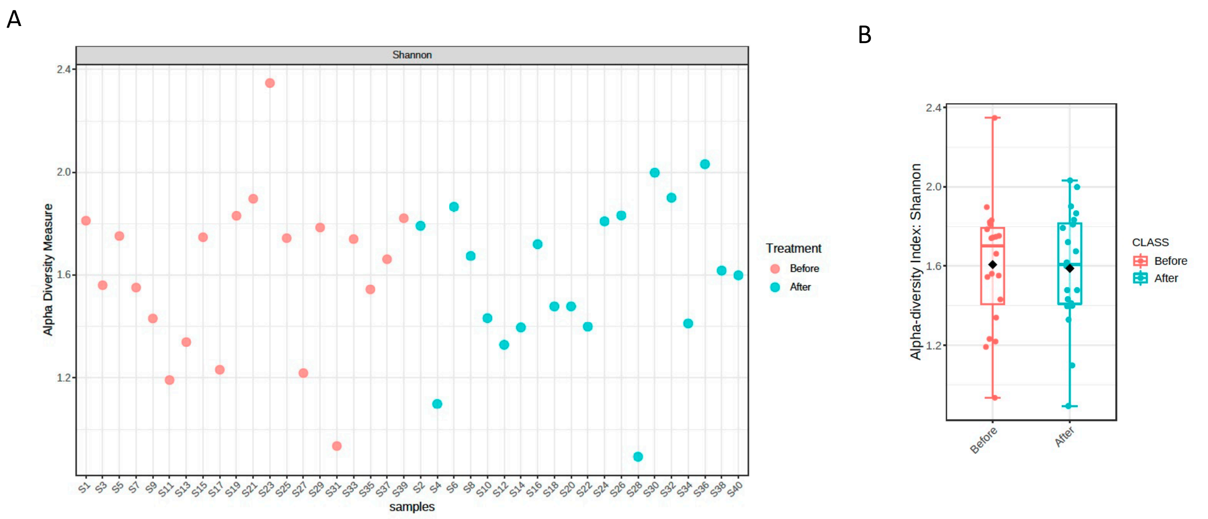

Figure S2. Alpha-diversity measure using Shannon at Genus level across all the samples. A - The samples are represented on X-axis and their estimated diversity on Y-axis, each sample is colored based on Treatment class; B – Box plot presentation, each boxplot represents the diversity distribution of a group present within Treatment class.
